# The Health Officer of New York

**Published:** 1880-02

**Authors:** 


### THE HEALTH OFFICER OF NEW YORK.

In the proceedings of the Erie County Medical Society, published elsewhere, will be found appropriate action, endorsing the name of Prof. Julius F. Miner, of this city, for the important position of Health Officer of the Port of New York. The unanimity with which the profession joins in urging the appointment of our late editorial confrere, and the universal favor with which the announcement of his candidacy is received, should impart an impetus to the movement thus inaugurated, which we hope will result in the recognition both of the claims of the distinguished individual, whose name is thus prominently brought forward, and of the section of the State of which he is the honored representative.

We but echo the sentiment of the entire profession, in stating that Prof. Miner's eminent fitness for the position, not only as to professional attainments, enlarged experience, but also, as to administrative ability, will reflect great credit upon the wise judgment of Gov. Cornell, should he bestow this position now at his disposal, upon one so peculiarly adapted to perform its responsible duties.

